# Impact of Doxorubicin on Cardiac Function in Dogs: Ejection Fraction Changes and Heart Failure Risk

**DOI:** 10.1002/vms3.70497

**Published:** 2025-08-25

**Authors:** Gustavo Cavinato Herrera, Luiz Ricardo Soldi, Leandro Machado Oliveira, Luiz Renato Paranhos, Marcelo José Barbosa Silva

**Affiliations:** ^1^ Institute of Biomedical Science Federal University of Uberlândia—UFU Uberlândia Minas Gerais Brasil; ^2^ Oral Sciences/Periodontics at Universidade Federal de Santa Maria Santa Maria Brasil; ^3^ School of Dentistry Federal University of Uberlândia—UFU Uberlândia Minas Gerais Brasil

**Keywords:** doxorubicin, echocardiography, ejection fraction

## Abstract

Doxorubicin is an antitumor antibiotic. It is often used in veterinary medicine to treat and extend the lives of dogs with cancer. A cardiotoxic side effect can lead to heart failure and treatment discontinuation. This systematic review and meta‐analysis aimed to evaluate the drug's cardiotoxic effects on the ejection fraction (EF) of dogs in doxorubicin protocols. The search was done in eight databases, with a total of 3587 articles screened, resulting in fifteen eligible articles included. A report on the included studies’ methods and results was done. It also assessed the risk of bias. Thirteen articles demonstrated cardiac changes in echocardiography with different routes of administration (intravenous and intracoronary). The intracoronary route was more toxic, and in all six studies performed, there was heart failure. The intravenous route caused heart failure in six of the nine studies. A meta‐analysis showed this drug worsened heart disease. It included four studies where it significantly lowered the EF. Overall, the intervention produced a mean reduction of 21.24% in EF. This review shows doxorubicin's impact on cardiac function. It reveals the need for careful monitoring of each patient.

## Graphical abstract

Graphical abstract summarizing the impact of doxorubicin on cardiac function in dogs, including methodology, key findings and clinical implications. *Image generated by ChatGPT (OpenAI) based on the article data*. This study evaluated the effects of doxorubicin on cardiac function in dogs through a systematic review and meta‐analysis. Fifteen studies involving 189 dogs were included. Intracoronary administration caused heart failure in 100% of cases, whereas intravenous use led to failure in 67%. Ejection fraction was reduced by an average of 21.24%. Doxorubicin showed significant cardiotoxicity, highlighting the need for continuous echocardiographic monitoring. Less toxic alternatives, such as modern anthracyclines or cardioprotective agents, should be considered in canine cancer treatment.

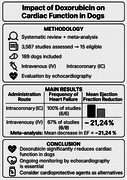

## 1 Introduction

The prevalence of cancer in veterinary medicine has been increasing considerably, a fact associated with the increase in life expectancy of animals, improvements in nutrition and increased diagnostic capacity due to the technological development of complementary exams and greater specialization of professionals (Nardi et al. 2002). Chemotherapy is used in the routine of veterinary cancer patients, with cytotoxic drugs used for the goal of complete remission of systemic neoplasms, such as lymphoma, reduction of tumours and increase in life expectancy and quality of life of animals with metastatic cancers (Macdonald 2009).

Malignant neoplasms such as lymphoma, osteosarcoma, and carcinomas respond to doxorubicin treatment, an antitumor antibiotic widely used in veterinary medicine (Hallman et al. 2019). This drug generates the formation of free radicals that can damage the cell membrane and DNA, altering their function (Pereira Neto et al. 2006). The cardiotoxicity of doxorubicin has been reported in several species, including humans. This effect has been described as dose‐dependent, with treatment being recommended in some cases or contraindicated in others (Costa et al. 2008), because doxorubicin has the potential to cause myocardial injury, leading to necrosis of the cardiac muscle with consequent loss of systolic function and dilation of the chambers, with these changes being observed through Doppler echocardiography.

Echocardiography is a non‐invasive imaging diagnostic method capable of measuring the diameter of the heart chambers, the thickness of the muscles and valves, and evaluating haemodynamic aspects and systolic and diastolic functions (Boon 2011). This test is widely used in veterinary medicine, and reference values for the various weight ranges and breeds of dogs have been described. Symptomatic cardiotoxicity in dogs treated with doxorubicin is not a consensus, and the dose used, the residual effect, and the patient's condition are factors that influence the presence of these signs. Thus, this systematic review aims to analyse the experimental studies that treated dogs with doxorubicin and identify the main echocardiographic changes observed in the studies.

## 2 Methods

### 2.1 Registration and Protocol

The project was initially registered with the Open Science Framework (OSF) (registration available at: https://osf.io/esa4k/) to promote transparency and reproducibility of the study. This systematic review followed the Preferred Reporting Items for Systematic Review and Meta‐Analysis Protocols (PRISMA‐P) (Page et al. 2021) and was conducted following the Joanna Briggs Institute Manual (JBI) (JBI Manual for Evidence Synthesis).

### 2.2 Question and Eligibility Criteria

This systematic review was conducted to answer the question: ‘What are the echocardiographic alterations found in dogs undergoing doxorubicin treatment?’, following the PVO (population, variables and outcomes) structuring strategy. The strategy developed consists of (1) population: dogs; (2) variable: doxorubicin treatment and (3) outcomes: echocardiographic parameters.

#### 2.2.1 Inclusion Criteria

Studies that performed doxorubicin treatment in dogs were included. This inclusion considered studies that administered doxorubicin in isolation, allowing direct evaluation of its effects. Among these studies, those that presented echocardiographic evaluation before, during and after treatment were included, allowing a longitudinal study of each case. Both prospective randomized and non‐randomized studies were considered. No date restriction was applied to this systematic review and only articles in language of Anglo‐Saxon or Latin origin were included.

#### 2.2.2 Exclusion Criteria

Review articles, editorials, letters and opinion pieces were excluded. Studies lacking echocardiographic data before, during, and after treatment were also excluded, as these data are essential to assess doxorubicin's cardiotoxicity accurately. Pre‐existing heart conditions could affect results, and the cumulative effect of doxorubicin is a major contributor to cardiotoxicity. Finally, studies without a control group in tests involving other drug combinations and articles in languages other than Latin or Anglo‐Saxon were excluded.

### 2.3 Sources of Information, Research, and Selection of Studies

Electronic searches were performed in the following databases: BMC, Embase, Lilacs, Livivo, Medline (PubMed) and the VHL Regional Portal. For the search in the gray literature, Google Scholar and Open Access Theses and Dissertations (OATD) were used.

Search strategies with Boolean markers ‘AND’ and ‘OR’ were performed, respecting the syntax rules of each database (Table 1). The results obtained were exported to EndNote WebTM software (Clarivate Analytics, Philadelphia, USA), in which duplicates were automatically removed. The remaining results were exported to the online tool Rayyan QCRI (Qatar Computing Research Institute, Doha, Qatar) for the study screening phase.

**TABLE 1:** Strategies for databases search.

Database	Search strategy
BMC veterinary	‘Dogs’ OR ‘Canine’ AND ‘doxorrubicin’ OR ‘anthracycline’ AND ‘cardiotoxicity’ OR ‘cardiac disease’ OR ‘cardiomiopathy’ OR ‘left ventricular function’ AND ‘Echocardiogram’ OR ‘Echodopplercardiogram’
Embase	(‘Dogs’/exp OR ‘dogs’ OR ‘canine’/exp OR ‘canine’) AND (‘chemotherapy’/exp OR ‘chemotherapy’ OR ‘doxorrubicin’ OR ‘adriamycin’/exp OR ‘adriamycin’ OR ‘anthracycline’/exp OR ‘anthracycline’) AND (‘echocardiogram’/exp OR ‘echocardiogram’ OR ‘echodopplercardiogram’)
Lilacs	(‘Dogs’ OR ‘canine’) AND (‘doxorrubicin’ OR ‘anthracycline’) AND (‘cardiotoxicity’ OR ‘cardiac disease’ OR ‘cardiomiopathy’ OR ‘left ventricular function’)
Livivo	‘Dogs’ OR ‘canine’ AND ‘doxorrubicin’ OR ‘anthracycline’ AND ‘cardiotoxicity’ OR ‘cardiac disease’ OR ‘cardiomiopathy’ OR “left ventricular function’ AND ‘echocardiogram’ OR ‘echodopplercardiogram’ dogs
Medline‐PubMed	‘Dogs’ OR ‘canine’ AND ‘doxorrubicin’ OR ‘anthracycline’ AND ‘cardiotoxicity’ OR ‘cardiac disease’ OR ‘cardiomiopathy’ OR ‘left ventricular function’ AND ‘echocardiogram’ OR ‘echodopplercardiogram’ OR ‘echodopplercardiography’
Portal regional da BVS	(‘Dogs’ OR ‘canine’ AND ‘doxorrubicin’ OR ‘anthracycline’) AND (‘cardiac disease’)
**Gray literature**	
Google scholar	‘Dogs’ OR ‘canine’ AND ‘doxorrubicin’ OR ‘anthracycline’ AND ‘cardiotoxicity’ OR ‘cardiac disease’ OR ‘cardiomiopathy’ OR ‘Left ventricular function’ AND ‘echocardiogram’ OR ‘echodopplercardiogram’
OATD	([‘Dogs’ OR ‘Canine’] AND [‘doxorrubicin’ OR ‘anthracycline’] AND [cardiomiopathy])

Abbreviation: OATD, Open Access Theses and Dissertations.

Two reviewers systematically analysed all selected articles in three phases. In the first phase, two eligibility reviewers (GCH and LRS) methodically analysed the titles, followed by the abstracts of the studies. Disagreements between examiners were analysed, and the decision was made by a third examiner (MJBS). In the first phase, articles unrelated to the topic, studies with other species, and abstracts that did not meet the eligibility criteria were excluded. In the third phase, full texts of the preliminary eligible studies were obtained and evaluated. Articles that did not present the echocardiographic results before, during and after treatment with doxorubicin were excluded in this phase as well.

### 2.4 Assessment of Sponsorship Status

To assess the risk of bias in the studies included in this review, the sources of sponsorship were investigated, as studies sponsored by pharmaceutical companies may be favourable to the product or drug compared to other sponsoring sources. To classify the sponsorship status, the following definition was used: unclear: articles that did not contain sponsorship information, making it impossible to identify whether they were sponsored; not sponsored: articles in which the authors declare that they do not have financial support; and sponsored: articles that contained statements by the authors of financial support from pharmaceutical companies.

### 2.5 Risk of Bias of the Studies

Using the JBI Systematic Reviews tool for randomized clinical trials (RCT) and uncontrolled clinical trials (UCT) (JBI Manual for Evidence Synthesis), the risk of bias in each article was assessed. Each question evaluated was classified as ‘yes’ when there was no bias for the question, ‘no’ if the study was biased for the question, ‘unclear’ when the information was not sufficient to answer that question and ‘not applicable’ when the question was not appropriate for that study.

### 2.6 Data Analysis

Data were summarized in evidence tables to determine study characteristics. Differences in EF were analysed using a pair‐wise meta‐analysis format. The results for FS were not suitable for meta‐analysis due to significant methodological heterogeneity. When change scores were not reported, they were calculated by subtracting the final from baseline scores and estimating the change in standard deviation (SD) using the formula SD_delta_ = √[(SD_baseline_)^2^ + (SD_final_)^2^ − (2 × *r* × SD_baseline_ × SD_final_)], in which we assumed *r* = 0.5 (Higgins et al. 2019). The mean difference (MD) was defined along with 95% confidence intervals (CI) as a summary estimate. All tests were two‐tailed, with a significance level set at 0.05—except Cochran's *Q* test (significance level at 0.1) which was used to assess the presence of heterogeneity and quantified it using the *I* ^2^ statistic, with values of 75%–100% indicating high heterogeneity. High heterogeneity was anticipated; therefore, a random‐effects model with the DerSimonian and Laird variance estimator was used (DerSimonian and Laird 1986). Prediction intervals for pooled MDs were estimated to provide a range of expected effects (Borenstein et al. 2017). Heterogeneity was explored through subgroup analyses according to the cumulative dose. All analyses were performed using Stata, version 14.0 (Stata Corporation).

As fewer than 10 studies were included in this meta‐analysis, formal quantification of publication bias and assessment of heterogeneity using meta‐regression were not performed.

## 3 Results

### 3.1 Study Selection

**FIGURE 1:**
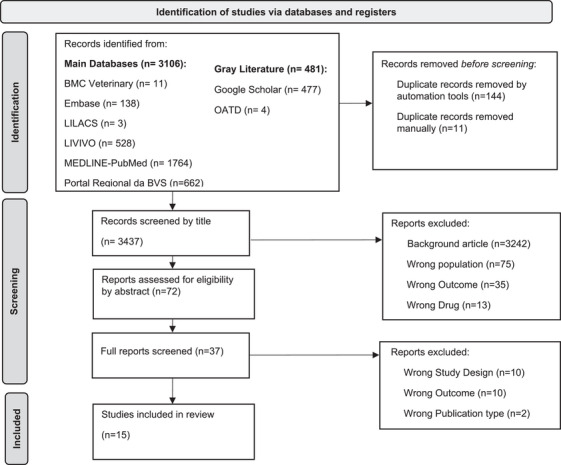
Database search and record selection flow diagram. PRISMA 2020 flow diagram for new systematic reviews which included searches of databases, registers and other sources. OATD, Open Access Theses and Dissertations. *Source*: Page et al. (2021). https://doi.org/10.1136/bmj.n71. For more information, visit: http://www.prisma‐statement.org/.

**FIGURE 2:**
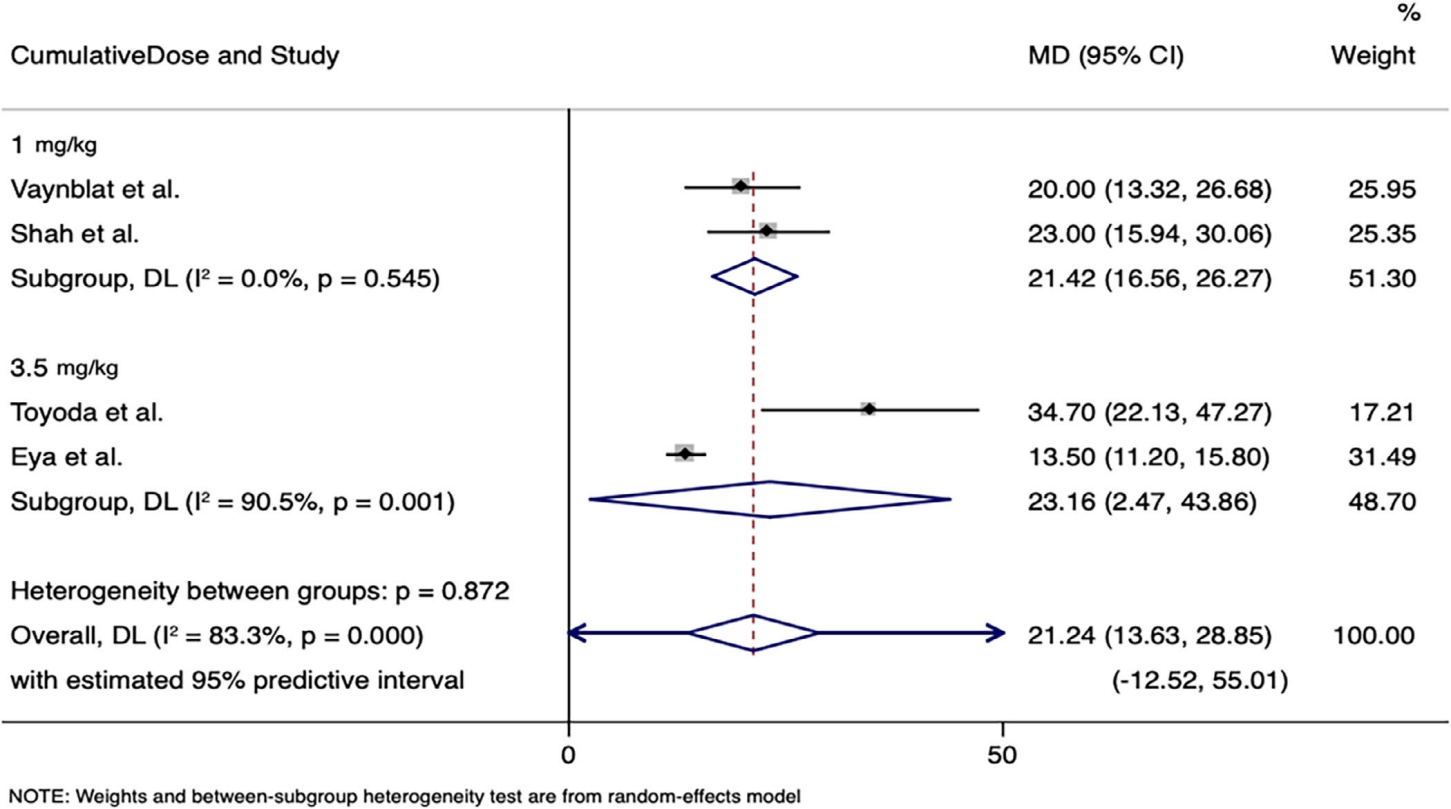
Meta‐analysis of the included studies. CI, confidence intervals; MD, mean difference.

An electronic search across eight databases, including gray literature, generated a total of 3587 results. After removing 150 duplicates—144 electronically and 11 manually—3364 articles were excluded on the basis of the title review. Following a thorough evaluation of the abstracts, an additional 34 articles were eliminated, leaving 37 articles for qualitative assessment. Ultimately, 15 studies met the established eligibility criteria and were included in this systematic review (Figure 1) (Styles et al. 1983; Unverferth et al. 1985; Hanai et al. 1996; Vaynblat et al. 1997, 2002; Shah et al. 1997; Toyoda et al. 1998; Christiansen et al. 2002; Eya et al. 2005; Alves de Souza and Camacho 2006; Sousa et al. 2014; Tater et al. 2017; Beaumier et al. 2020; Matsuura et al. 2021; Surachetpong et al. 2016) (Figure 2).

### 3.2 Characteristics of the Included Studies

The articles were published between 1983 and 2023 and conducted in six different countries: five in the United States of America, three in Germany, two in Japan, two in Brazil, one in Canada, and one in Thailand. According to the experimental design, nine studies were randomized and seven were non‐randomized. In the 15 eligible studies, 189 dogs participated in the research.

Two different doxorubicin route applications were assessed: intravenous (nine studies) and intracoronary (six studies). A dose of 1.5 mg/kg was used in one study using the intravenous route; in the other eight studies, the dose was 1 mg/kg (30 mg/m^2^). The dose administered intracoronarily ranged from 0.25 mg/kg (three studies), 0.7 mg/kg (two studies) and 10 mg/animal (mean of 0.30 mg/kg) (one study). Applications were administered weekly or at 3‐week intervals, with treatment duration ranging from 3 to 12 applications.

### 3.3 Individual Study Results

Of the 15 articles eligible for this systematic review, 13 reported cardiac changes after doxorubicin administration. Decreased function was the main change found, with the shortening fraction (FS) and ejection fraction (EF) as variables, representing impaired cardiac muscle contractility, reported in 100% of the studies with echocardiographic changes. The increase in the internal diameter of the heart, described in 10 articles, appears to be another important variable. The intracoronary route for administration of doxorubicin was shown to be more toxic, and in all six studies performed, there was cardiac dilation with decreased function. The intravenous route caused heart failure in six of the nine studies. The main echocardiographic findings are shown in Table 2.

**TABLE 2:** Characteristics of eligible studies.

References	Sample size	DOXO administration	Dose	Echocardiographic findings	EF		FS		Total cumulative dose
Styles et al. (1983)	9	Intra venous infusions	1 mg/kg	↓function	↓24%	75–57	—	—	12 mg/kg
Unverferth et al. (1985)	38	Intra venous infusions	1 mg/kg	↓function, ↑internal diameter	—	—	↓38%	—	12 mg/kg
Hanai et al. (1996)	12	Intra venous infusions	1.5 mg/kg	↓function	—	—	↓65%	50—16	10.5 mg/kg
Vaynblat et al. (1997, 2002)	10	Intracoronary	0.25 mg/kg	↓function, ↑internal diameter	↓33%	60—40	—	—	1 mg/kg
Shah et al. (1997)	20	Intracoronary	0.25 mg/kg	↓function, ↑internal diameter	↓39%	60—37	—	—	1 mg/kg
Toyoda et al. (1998)	9	Intracoronary	0.7 mg/kg	↓function, ↑internal diameter	↓43%	71–36.3	↓40%	36.5–21.7	3.5 mg/kg
Christiansen et al. (2002)	6	Intracoronary	10 mg/animal	↓function, ↑internal diameter	↓50%	61.1–30.5	↓33%	41.7–27.9	40 mg/animal
Vaynblat et al.	17	Intracoronary	0.25 mg/kg	↓function, ↑internal diameter	↓25%		—	—	1 mg/kg
Eya et al. (2005)	7	Intracoronary	0.7 mg/kg	↓function, ↑internal diameter	↓37%	35.5–22	—	—	3.5 mg/kg
Alves de Souza and Camacho (2006)	13	Intra venous infusions	30 mg/m^2^	↓function, ↑internal diameter	↓45%	66–36	↓55%	36–17	240 mg/m^2^
Sousa et al. (2014)	7	Intra venous infusions	30 mg/m^2^	↓function, ↑internal diameter	↓36.5%	66.7–42	↓47.5%	36.1–19.1	210 mg/m^2^
Surachetpong et al. (2016)	12	Intra venous infusions	30 mg/m^2^	↓function	↓13%	62.21–54.74	↓30.5%	36.06–25.27	120 mg/m^2^
Tater et al. (2017)	14	Intra venous infusions	30 mg/m^2^	No echocardiographic changes	—	—	—		120 mg/m^2^
Beaumier et al. (2020)	9	Intra venous infusions	30 mg/m^2^	↓function, ↑internal diameter	↓25.7%	70.74–60.67	↓14.3%	38.91–28.9	120 mg/m^2^
Matsuura et al. (2021)	6	Intra venous infusions	30 mg/m^2^	No echocardiographic changes	(40–34) *p* 0.13	—	(78–70) *p* 0.15	—	180 mg/m^2^

Abbreviation: EF, ejection fraction.

### 3.4 Meta‐Analysis

The meta‐analysis included four studies, with a total of 79 animals. Overall, the intervention produced a mean reduction of 21.24% in EF (95% CI: 13.63%, 28.85%; *I* ^2^ = 83.3%). When stratifying the analysis according to the cumulative dose, the mean reductions in EF levels were similar; however, heterogeneity in the 3.5 mg/kg subgroup was important (*I* ^2^ = 90.5%). We expect that in some 95% of intervals of all populations, the true MD falls in the approximate range of −12.52% to 55.01% (Figure 2).

### 3.5 Risk of Bias for Each Study

Information on the risk of bias is shown in Table 3. The articles were classified as RCT and quasi‐experimental studies. Overall, most studies presented a low risk of bias, one study a high risk, and six a moderate risk.

**TABLE 3:** Risk of bias.

Authors, (year)	Type of study	Q1	Q2	Q3	Q4	Q5	Q6	Q7	Q8	Q9	Q10	Q11	Q12	Q13	% of Yes	Risk of bias
Styles et al. (1983)	RCT	—	—	✓	—	—	—	✓	✓	✓	✓	✓	✓	✓	61.53	Moderate
Unverferth et al. (1985)	RCT	✓	—	✓	—	—	—	✓	✓	✓	✓	✓	✓	✓	69.23	Moderate
Hanai et al. (1996)	RCT	—	—	✓	—	—	—	✓	✓	✓	✓	✓	✓	✓	61.53	Moderate
Vaynblat et al. (1997)	RCT	—	✓	✓	—	—	—	✓	✓	✓	✓	✓	✓	✓	69.23	Moderate
Shah et al. (1997)	Quasi‐experimental	✓	✓	—	—	✓	✓	✓	✓	✓	N/A	N/A	N/A	N/A	77.77	Low
Toyoda et al. (1998)	RCT	U	—	✓	—	—		✓	✓	✓	✓	✓	✓	✓	61.53	Moderate
Christiansen et al. (2002)	Quasi‐experimental	✓	✓	—	—	✓	✓	✓	✓	✓	N/A	N/A	N/A	N/A	77.77	Low
Vaynblat et al. (2002)	RCT	✓	—	✓	—	—	—	✓	✓	✓	✓	✓	✓	✓	69.23	Moderate
Eya et al. (2005)	Quasi‐experimental	✓	✓	—	—	✓	✓	✓	✓	✓	N/A	N/A	N/A	N/A	77.77	Low
Alves de Souza and Camacho (2006)	RCT	✓	—	✓	—	—	✓	✓	✓	✓	✓	✓	✓	✓	76.92	Low
Sousa et al. (2014)	Quasi‐experimental	✓	✓	—	—	✓	✓	✓	✓	✓	N/A	N/A	N/A	N/A	77.77	Low
Surachetpong et al. (2016)	RCT	—	—	—	—	—	—	—	✓	✓	✓	✓	✓	✓	46.15	High
Tater et al. (2017)	Quasi‐experimental	✓	✓	—	—	✓	✓	✓	✓	✓	N/A	N/A	N/A	N/A	77.77	Low
Beaumier et al. (2020)	Quasi‐experimental	✓	✓	—	—	✓	✓	✓	✓	✓	N/A	N/A	N/A	N/A	77.77	Low
Matsuura et al. (2021)	Quasi‐experimental	✓	✓	—	—	✓	✓	✓	✓	✓	N/A	N/A	N/A	N/A	77.77	Low

*Note*: ✓—yes; —No; U—unclear; randomized studies: ‘Q1. Was true randomization used for assignment of participants to treatment groups? Q2. Was allocation to treatment groups concealed? Q3. Were treatment groups similar at the baseline? Q4. Were participants blind to treatment assignments? Q5. Were those delivering treatment blind to treatment assignment? Q6. Were outcomes assessors blind to treatment assignment? Q7. Were treatment groups treated identically other than the intervention of interest? Q8. Was follow up complete and if not, were differences between groups in terms of their follow up adequately described and analysed? Q9. Were participants analysed in the groups to which they were randomized? Q10. Were outcomes measured in the same way for treatment groups? Q11. Were outcomes measured in a reliable way? Q12. Was appropriate statistical analysis used? Q13. Was the trial design appropriate, and any deviations from the standard RCT design (individual randomization, parallel groups) accounted for in the conduct and analysis of the trial?’; Quasi experimental studies—(Q1) ‘is it clear in the study what is the cause’ and what is the ‘effect’ (i.e., there is no confusion about which variable comes first)?’ (Q2) ‘Were the participants included in any comparisons similar?’ (Q3) ‘Were the participants included in any comparisons receiving similar treatment/care, other than the exposure or intervention of interest?’ (Q4) ‘Was there a control group?’ (Q5) ‘Were there multiple measurements of the outcome both pre and post the intervention/exposure?’ (Q6) ‘Was follow up complete and if not, were differences between groups in terms of their follow up adequately described and analysed?’ (Q7) ‘Were the outcomes of participants included in any comparisons measured in the same way?’ (Q.8) ‘Were outcomes measured in a reliable way?’ (Q9) ‘Was appropriate statistical analysis used?’.

Abbreviations: √, yes; —, no; U, unclear; N/A, not applicable, RCT: randomized control study.

### 3.6 Assessment of the Sponsorship Status of the Studies

Table 4 presents the results of the assessment of the sources of sponsorship of the studies included in this review. In eight articles, the sources of sponsorship were research institutes; in the remaining seven studies, clear information on the sponsorship status was not declared. No studies declared sponsorship by pharmaceutical companies.

**TABLE 4:** Sponsorship status of the studies.

References	Sponsorship status	Sponsorship
Styles et al. (1983)	Sponsored	Canadian Cancer Society Alberta Cancer Board, Edmonton, Alberta
Unverferth et al. (1985)	Unclear	
Hanai et al. (1996)	Unclear	
Vaynblat et al. (1997, 2002)	Sponsored	Laboratories of Columbus, OH Gore & Associates Inc, of Flagstaff, AZ
Shah et al. (1997)	Sponsored	The Foundation for Surgical Education and Investigation Inc., SUNY‐HSC at Brooklyn The Maimonides Research and Development Foundation Adria Laboratories, Columbus, Ohio, for graciously providing our laboratory with doxorubicin
Toyoda et al. (1998)	Unclear	
Christiansen et al. (2002)	Unclear	
Vaynblat et al. (1997, 2002)	Sponsored	The Foundation for Surgical Education and Investigation Inc., SUNY‐HSC at Brooklyn The Maimonides Research and Development Foundation Adria Laboratories, Columbus, Ohio, for graciously providing our laboratory with doxorubicin
Eya et al. (2005)	Unclear	
Alves de Souza and Camacho (2006)	Sponsored	Fundação de Amparo à Pesquisa do Estado de São Paulo Royal Canin
Sousa et al. (2014)	Unclear	
Surachetpong et al. (2016)	Sponsored	TRF Grant for New Researcher, the Thailand Research Fund
Tater et al. (2017)	Unclear	
Beaumier et al. (2020)	Sponsored	Tufts University Companion Health Fund Barkley Fund and Shipley Foundation
Matsuura et al. (2021)	Sponsored	Japan Society for the Promotion of Science (JSPS)

## 4 Discussion

Doxorubicin, an anthracycline widely used in antineoplastic treatment in dogs, is known for its cardiotoxic effect, causing cellular damage that leads to cardiac dilation and loss of function (Surachetpong et al. 2016). Haemodynamic echocardiography is safe and can detect cardiac injury caused by cardiotoxic medications (Hanton et al. 2008). EF, which is characterized by the percentage of blood ejected by the left ventricle in each cardiac cycle, is an echocardiographic parameter used to measure myocardial function and was shown to be reduced in 11 of the 15 studies evaluated. This finding corroborates the importance of its evaluation in patients undergoing chemotherapy protocols with this drug. Doxorubicin is known to cause several forms of cardiac injury, including myocardial fibrosis, atrophy, and myocyte lysis (Mauldin et al. 1992). This damage leads to stiffening of the cardiac muscle fibre, reducing its capacity and number. This decrease in both the functionality and quantity of heart muscle cells diminishes contractility, which is often measured by a decrease in BF. which was observed in eight of the 15 studies analysed in this review. This finding of reduced BF was consistent across eight of the 15 studies analysed in this review.

Antineoplastic antibiotics act on cell DNA, inhibiting its replication, and their main drugs are doxorubicin, mitoxantrone, actinomycin, bleomycin and epirubicin. Due to differences in price and effectiveness, there is a need for studies that evaluate and compare these drugs. Mitoxantrone, for example, has a mechanism of action and effectiveness like doxorubicin albeit with milder side effects when compared (Franco et al. 2019). One study compared the cytotoxic effects of mitoxantrone with doxorubicin, with the former presenting no evidence of cardiac changes while the latter did, though gastrointestinal effects and myelosuppression were observed with mitoxantrone (Henderson et al. 1989). Its use in veterinary medicine is still limited due to its high cost, which can be up to four times higher when compared to doxorubicin. Being an available alternative, more studies with this drug must be carried out, making it a valid option, especially for patients with previous heart disease.

In the included studies that used the intracoronary route, all dogs developed heart failure, as expected, as the objective was to evaluate this outcome (Vaynblat et al. 1997, 2002; Shah et al. 1997; Toyoda et al. 1998; Christiansen et al. 2002; Eya et al. 2005). The greater toxicity can be explained by the higher concentration of the drug in the heart muscle as the application is made directly through the implantation of specific devices. The use of the intravenous (IV) route carried out for antineoplastic treatment also presented cardiotoxic potential, with doses above 30 mg/m^2^ not being recommended, nor cumulative doses above 250 mg/m^2^, which increases the incidence of heart failure (Pereira Neto et al. 2006). In six articles included in this review, the intravenous was the route for doxorubicin administration, and in four studies, there was heart disease. In one study, cumulative doses of 90 mg/m^2^ and an interval between applications of 3 weeks were sufficient to demonstrate cardiotoxicity (Alves de Souza and Camacho 2006), demonstrating that, even though the intracoronary route is more cardiotoxic, the therapeutic doses used for antineoplastic treatment (IV) may be sufficient to generate heart disease.

The four studies evaluated in this meta‐analysis showed a decrease in EF and, consequently, a decrease in cardiac function (Vaynblat et al. 1997, 2002; Shah et al. 1997; Toyoda et al. 1998; Eya et al. 2005). The degree of decreased function was similar even when comparing different cumulative doses (1.0 and 3.5). In a meta‐analysis study carried out by Jeyaprakash et al., the average reduction for EF was 5.4%. This meta‐analysis included various anthracyclines, such as doxorubicin and epirubicin, highlighting the cardiotoxic potential of this class of drugs and the importance of individual assessment of each patient before including them in oncological treatments, as pre‐existing heart disease can favour the appearance of heart damage, and this drug is contraindicated for individuals with severe heart disease.

The studies carried out by Tater et al. (2017) and Matsuura et al. (2021) evaluated patients undergoing cancer treatment and healthy patients, respectively. Their finding did not prove significant echocardiographic changes as a decrease in EF and FS, even in different research conditions. These data reinforce that doxorubicin is still a viable treatment option for canine cancer patients, given its already proven effectiveness and low cost for those responsible. Additionally, the use of cardioprotectors in human patients reduces the harmful effects, by reducing the oxidative stress of cardiac cells through the removal of free radicals. The administration of rosuvastatin, for example, significantly reduced the cardiotoxic effect of doxorubicin (Kettana et al. 2024) proving to be an option for further studies in dogs.

The included studies were classified as randomized and quasi‐experimental according to the model adopted. The differences between the models resulted from different approaches such as the use of animals from routine veterinary oncology care, evaluation of the cardioprotective effect of drugs applied in conjunction with doxorubicin, and studies that aimed to generate heart failure for different subsequent treatments. These differences in study design resulted in inconsistencies across the studies, such as a lack of standardized groups or even the absence of a control group in some cases. This lack of standardization posed challenges for pairing studies and conducting meaningful statistical analyses.

A key limitation of this systematic review is the small sample size in most of the included studies, except for Unverferth et al. (1985), which evaluated 38 animals. Furthermore, the use of varying echocardiographic parameters across the studies hindered statistical comparisons.

In conclusion, future research should prioritize larger sample sizes and standardize echocardiographic variables to facilitate a more robust evaluation of doxorubicin‐induced heart failure. Despite its established cardiotoxicity, doxorubicin remains a valuable antineoplastic drug in canine cancer treatment due to its efficacy. This review confirms the impact of doxorubicin on cardiac function, revealing the critical importance of comprehensive monitoring, including pre‐, intra‐ and post‐chemotherapy assessments, along with individualized risk evaluation for each patient. Additionally, the use of modern anthracyclines may offer a less cardiotoxic alternative, enhancing the safety of future treatments of canine cancer patients.

## Author Contributions

**Gustavo Cavinato Herrera**: conceptualization, methodology, formal analysis, writing – original draft, writing – review and editing, visualization. **Luiz Ricardo Soldi**: conceptualization, methodology, formal analysis, writing – original draft, writing – review and editing, visualization. **Leandro Machado Oliveira**: methodology, formal analysis, data curation. **Luiz Renato Paranhos**: methodology, writing – review and editing. **Marcelo José Barbosa Silva**: conceptualization, methodology, writing – review and editing, project administration, supervision. All authors contributed to the article and approved the submitted version.

## Ethics Statement

The authors have nothing to report.

## Conflicts of Interest

The authors declare no conflicts of interest.

## Peer Review

The peer review history for this article is available at https://www.webofscience.com/api/gateway/wos/peer‐review/10.1002/vms3.70497.

## Data Availability

The original contributions presented in the study are included in the article, and further inquiries can be directed to the corresponding author.
